# The Chemical and Products Database, a resource for exposure-relevant data on chemicals in consumer products

**DOI:** 10.1038/sdata.2018.125

**Published:** 2018-07-10

**Authors:** Kathie L. Dionisio, Katherine Phillips, Paul S. Price, Christopher M. Grulke, Antony Williams, Derya Biryol, Tao Hong, Kristin K. Isaacs

**Affiliations:** 1 U.S. Environmental Protection Agency, National Exposure Research Laboratory, 109 T.W. Alexander Drive, Research Triangle Park, NC 27709, USA; 2 U.S. Environmental Protection Agency, National Center for Computational Toxicology, 109 T.W. Alexander Drive, Research Triangle Park, NC 27709, USA; 3 Oak Ridge Institute for Science and Education, Oak Ridge, TN 37830, USA; 4 ICF International, 2635 Meridian Pkwy #200, Durham, NC 27713, USA

**Keywords:** Public health, Toxicology, Environmental social sciences

## Abstract

Quantitative data on product chemical composition is a necessary parameter for characterizing near-field exposure. This data set comprises reported and predicted information on more than 75,000 chemicals and more than 15,000 consumer products. The data’s primary intended use is for exposure, risk, and safety assessments. The data set includes specific products with quantitative or qualitative ingredient information, which has been publicly disclosed through material safety data sheets (MSDS) and ingredient lists. A single product category from a refined and harmonized set of categories has been assigned to each product. The data set also contains information on the functional role of chemicals in products, which can inform predictions of the concentrations in which they occur. These data will be useful to exposure and risk assessors evaluating chemical and product safety.

## Background & Summary

Evaluating chemical safety and sustainability over the life cycle of products requires drawing upon the various data streams and impact assessment tools from the life cycle assessment (LCA) field, along with improved exposure models that rapidly and reliably characterize human health risks of chemicals from direct and indirect exposure pathways. Near-field exposure to chemicals in consumer products has been identified as a significant source of exposure for many chemicals^1,2^. Quantitative data on product chemical composition is a necessary parameter for characterizing near-field exposure.

Currently there are limited data on the composition of products available in a format which allows for large-scale modeling of near-field exposures to chemicals in consumer products. Previous efforts at the U.S. Environmental Protection Agency (U.S. EPA) have aggregated data on consumer product composition (the Chemical/Product Categories Database (CPCat)^3^, and the Consumer Product Chemical Profiles database (CPCPdb^4^)) and functional use of chemicals (i.e., the role a chemical plays in a product, e.g. solvent vs. fragrance; the Functional Use database (FUse)^5,6^). Here we describe the Chemicals and Products Database (CPDat), a data set of consumer product composition and functional use, including information on >75,000 chemicals and >15,000 consumer products. CPDat incorporates CPCat, CPCPdb, and FUse in full, and streamlines the three data sets with a consistent scheme for categorizing products and chemicals. In addition, CPDat includes seven distinct newly acquired data sets on product composition (both reported values and quantitative predictions based on ingredient list labels) and functional use. The newly acquired data sets have expanded the scope and number of records in the database which the user has to draw from, dependent on their data needs. Further, harmonization of the existing databases allows for a richer data set which describes the full extent of the chemical-product data relevant for exposure modeling of consumer products.

Each product-related record (i.e., a unique piece of data corresponding to a specific consumer product, e.g., an MSDS sheet for a particular product) in CPDat is linked to a unique product category. Product categories exist as a hierarchical, harmonized nomenclature for categorization of consumer products. Product records in CPDat contain chemical information, e.g., a product record may include the list of chemicals included in the formulation of that product. Each of these chemicals has been mapped to a unique, curated chemical record in the EPA’s Distributed Structure-Searchable Toxicity (DSSTox) Database, which underlies the publicly available CompTox Chemistry Dashboard (https://comptox.epa.gov/dashboard). The Dashboard is a resource which includes an abundance of information on the chemical’s structure, properties, and toxicology. All CPDat data are available via the ‘Exposure’ tab in the CompTox Dashboard, providing an easily accessible, central repository for product composition and functional use data.

**Figure 1:**
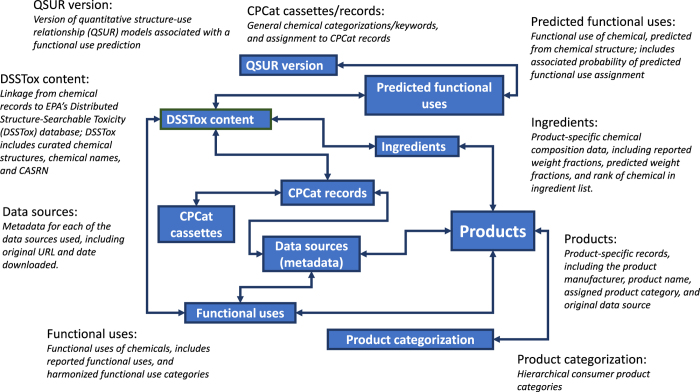
CPDat data structure.

As described in Fig. 1, CPDat currently includes the following types of data:

- Quantitative chemical composition
- Reported data on composition of a large number of consumer products, including weight fractions for each component, taken from publicly available Material Safety Data Sheets (MSDS)
- Predicted weight fractions of chemicals in consumer products, derived from publicly available ordered product ingredient lists and ingredient disclosures, which are documents that provide a list of the ingredients in a product, and the functional use that some or all of the ingredients serve in the product^7^
- Consistent product and chemical categorization
- General categorization of chemical usage (e.g. industrial uses, broad categorizations of use)^3^
- Consumer product category specific categorization
- Chemical functional use
- Reported chemical functional use data from publicly available government, manufacturer, and industry sources
- Reported data on functional uses of chemicals within consumer products from manufacturers
- Predicted data on functional use of chemicals, based on quantitative structure-use relationship (QSUR) modeling methods^6^

The data included in CPDat will inform exposure assessments of consumer products for commercial chemicals. Information about exposure is a critical component of risk evaluations and provides real-world context to hazard (i.e., toxicity data). These data can be reused in aggregate (i.e., single chemical, multi-product) and cumulative (i.e., multi-chemical) assessments by federal and state governments, academia, and industry.

## Methods

Data included in the CPDat data set represent an aggregation of publicly available data on chemical-use categorization, consumer product composition (including qualitative ingredient lists and quantitative composition data, i.e., weight fraction), and functional use of chemicals. All data included in CPDat were acquired or generated from publicly available sources as detailed below. CPDat represents the aggregation and harmonization of three existing data sets with newly available related data. Methods and included data sources presented here reflect the data set as of August 8, 2017. We anticipate that additional data will be added to the data set over time. The addition of new data will be documented in the public repository on both the ‘News’ (https://comptox.epa.gov/dashboard/news_info) and ‘Downloads’ (https://comptox.epa.gov/dashboard/downloads) pages of the Dashboard, as appropriate. The three existing data sets are described briefly below, with full details included in the associated publications. Additional details and methods are provided for the new data and sources included in CPDat.

### Curation of chemicals

Each data record in CPDat is associated with a chemical name and/or Chemical Abstracts Service Registry Numbers (CASRN). To enable integration of CPDat data with other DSSTox data and into the Dashboard, it was necessary to map CPDat chemicals to the appropriate DSSTox chemical identifier^8^. CPDat names and CASRN were searched against the complete list of potential chemical identifiers in DSSTox. Potential DSSTox generic substances for each CPDat chemical record were scored based on the quality of the automatically generated mapping, taking into account the trust associated with the various identifiers in the DSSTox database (e.g., a match against a validated synonym is scored higher than a match against an ambiguous synonym). A DSSTox generic substance with the top score was mapped to each CPDat chemical record.

### Existing datasets incorporated into CPDat

#### Chemical/Product Categories (CPCat) Database

The CPCat relational database was constructed from a variety of publicly available data sources on chemicals and associated categorical groupings. CPCat integrates information from 11 major national and international sources. Original data sources and methods are described in detail in Dionisio *et al.*^3^. A harmonized set of CPCat terms (general use keywords, sometimes but not always describing specific consumer products) and cassettes (unique keyword combinations) were manually linked to each chemical included in the data set, based on information on chemical usage available from the original source. Cassettes are comprised of one or more CPCat terms, all terms within a cassette must be interpreted together to reflect the categorical information provided by the original data source.

#### Consumer Product Chemical Profiles database (CPCPdb)

CPCPdb was the first effort to create a publicly available data set of consumer product composition and weight fractions to be used in high-throughput exposure modeling. The data set focused on MSDS available on the internet as Adobe PDF documents for a single retailer. Full details on methods used to extract data from the MSDS are available in Goldsmith *et al.*^4^. Briefly, MSDS were downloaded as PDF files, and programmatic tools including optical character recognition software were used to extract the product ingredient information. Data were stored in a MySQL database custom-designed for this purpose. Each entry was manually curated using a custom, web-enabled interface. Products were assigned to a product category and subcategory based on the categorization used on the retailer’s online shopping website, with the product categorization automatically retrieved from the retailer’s website using a freely available data extraction program.

#### Functional Use database (FUse)

FUse is a collection of chemicals in consumer products linked by the function (or role) that the chemical is known to have in products (e.g., fragrance, solvent, etc.). The database was created via automated collection of publicly available functional use data provided by manufacturers of consumer products, in ingredient repositories for product formulators, or from government regulators. The reported functional uses were harmonized to a set of consistent ‘root’ terms (e.g., ‘whitening agent’ and ‘whitener’ were mapped to the same term). For modelling purposes (functional use prediction), the functions were further harmonized using a hierarchical cluster analysis. A full description of the methods and compilation of FUse can be found in Isaacs*, et al.* and Phillips *et al.*^5,6^.

### Additional data

CPDat also includes data from the sources listed in Table 1. These data sources include product composition data (quantitative weight fraction) not included in previous databases curated by the authors, product-specific ingredient list data (qualitative), and functional use data. Data sources listed in Table 1 were all obtained from public internet sites of reputable manufacturers or retailers.

#### Proctor & Gamble

For the Proctor & Gamble data source, MSDS were automatically downloaded as PDF files and converted to TXT files using R scripts (http://www.R-project.org/). Composition data associated with each product were extracted from the TXT files automatically using custom Python (https://www.python.org/) scripts. This was possible because the MSDS followed a common format. If a chemical composition table spanned two pages, the words ‘Page xx’ were removed manually. Manual extraction of data was also required for Duracell products, which used a different MSDS template. Extracted data records were stored in CSV files.

#### Unilever MSDS USA

The PDF MSDS in the Unilever MSDS USA repository were downloaded manually, with data then extracted automatically using custom R and Python scripts as above, with extracted data stored in a CSV. For the Unilever MSDS USA data source, product type was available on the website, but not on the MSDS, thus this field was manually added to the data record, for use in assigning the product to a product category.

#### Proctor & Gamble Product Safety

The ingredient and functional use disclosures provided by Proctor & Gamble Product Safety were automatically downloaded as PDF files and converted to text files using the RCurl (http://CRAN.R-project.org/package=RCurl) and XML (http://CRAN.R-project.org/package=XML) packages for the R programming language. Pertinent information from the converted text files were parsed and stored in a CSV file via custom Python scripts. For some products, the name “fragrance” was listed as an ingredient with its functional use listed as a hyperlink to another PDF containing a list of chemicals used by the manufacturer as fragrances in products. In these cases, the hyperlink was removed and the functional use was replaced with “fragrance”.

#### Unilever

The ingredient disclosures provided by Unilever were obtained by downloading the HTML file of the disclosure for each product of each brand provided by the manufacturer. The ingredients and functional uses provided on the disclosures were listed in separate columns of an HTML table, which were parsed and stored in a CSV file. The files were automatically downloaded and parsed using the RCurl and XML packages for the R programming language. For some products, the ingredient disclosure was duplicated on the website (e.g., the HTML table contained records for twice the number of chemicals in a product, when in reality each chemical was listed with the same functional use twice in the table). Therefore, duplicated records of an ingredient-functional use pair were dropped from the parsed HTML table in such a way that ingredient order in the table was preserved.

#### Church & Dwight

Ingredient disclosures were automatically downloaded from the Church & Dwight website as PDF files using the RCurl and XML packages for the R programming language. PDFs were converted to text files which allowed for parsing of relevant information (i.e., product name, ingredient name, and ingredient functional use in product) into a CSV file via custom Python scripts.

#### Palmolive

All products and their ingredient disclosures were available in a single table from the Palmolive website. This information was manually copied and pasted into a text file which was then parsed into a CSV file via a custom Python script.

#### Drugstore.com

Reported ingredient data for products were obtained from Drugstore.com. Lists of links to HTML files for available products were obtained using custom R scripts employing the R packages RCurl and XML. The HTML file for each product was subsequently obtained and parsed using custom R text parsing scripts. The data were manually curated, with 2-component products identified. Each ingredient name and rank in the reported ingredient list were obtained for use in future weight fraction predictions. The product name and Drugstore.com product category was retained for use in harmonized product categorization.

#### Generated data fields

Data fields generated after data collection (assignment of product category, predicted weight fraction from ingredient lists, and harmonized and predicted functional use) are detailed below; all other data fields are replicated exactly as they were in the original data source.

For each product-specific record, the product was assigned to a product category. The product categories used are a refined set of categories based on those previously developed for use in linking products to exposure scenarios^9^, but further refined for product form (e.g., liquid versus spray cleaner) and population of use (e.g., children’s sunscreen). The product categories are reproduced in Table 2 (available online only). The assignment of products to categories was performed using the following process. Product names were first passed through a custom R script which performed a preliminary categorization of products that included a pre-identified list of key words (e.g. if product name included ‘shampoo’, assign product to ‘shampoo’ category). All product category assignments were then reviewed manually for accuracy. Assignment of product categories were completed manually for any product where a preliminary categorization was not possible.

Product-specific records linked to quantitative composition data were obtained in one of two ways. If the manufacturer provided quantitative composition data for a specific product, data were replicated in CPDat exactly as provided by the manufacturer. However, some sources included qualitative ingredient list data, providing a list of ingredients (by chemical name and/or CASRN) in the product without information on the weight fraction of each ingredient. When it was known that these ingredient lists were arranged in a specific order (e.g., personal care products, which are mandated by law to list ingredients in decreasing weight fraction order), a model detailed in ref. 7 was applied to the ingredient list to obtain the 5th and 95th percentile bounds on the weight fraction for each ingredient in the list.

Reported functional uses contained in FUse were harmonized across the various data sources. The harmonized functional use reported in CPDat is intended 1) to remove redundancy in functional uses reported by different sources (e.g.., “foaming aid” and “foam boosting agent” would be collapsed into “foam boosting agent”) and 2) to provide a single descriptive functional use for each chemical in FUse. For example, many chemicals are reported to have functional uses of both “cleanser” and “surfactant”, as cleansers are typically surfactants, these chemicals would have a single harmonized functional use of “surfactant”. The details of the harmonization process via hierarchical clustering have been previously described^5,6^. These harmonized uses were then used to train and validate a suite of quantitative structure-use relationship (QSUR) models that can predict a chemical’s likely functional use(s) for chemicals with no known functional use^5,6^. The full details of the development and validation of these models has been published previously^5,6^. Briefly, the models were developed from publicly-available structural descriptors using Random Forest classification and validated using 5-fold cross-validation and y-randomization^5,6^. For any chemical structure, the valid Random Forest model for a function returns the probability (0-1) of the chemical performing the given function. In CPDat, we report results from 41 valid function QSUR models for chemicals modelled in Phillips *et al*.^6^. The performance of these 41 valid models is provided in Table 3 (available online only).

### Code availability

Scripts to perform data downloads are available at: https://github.com/HumanExposure/CPDatManScripts.

## Data Records

The data repository where the CPDat consumer product and functional use data are stored, the U.S. EPA’s CompTox Chemistry Dashboard (hereafter referred to as the ‘Dashboard’), is available publicly at https://comptox.epa.gov/dashboard. The Dashboard is a freely accessible web-based application and data hub integrating data for ~760,000 chemical substances (as of August 2017), most of these with their related chemical structures. Various types of data are associated with the substances including both experimental and predicted physicochemical and environmental fate and transport properties, toxicity data, bioassay data (i.e. the ToxCast data^8^), exposure data and external links to relevant public data sources. When viewing a chemical’s landing page through the Dashboard, the CPDat data are available under the ‘Exposure’ tab, through the ‘Product & Use Categories,’ ‘Chemical Weight Fraction,’ and ‘Chemical Functional Use’ sub-tabs. Note that the CPDat data covers only a subset of the chemicals included in the Dashboard, but includes data on more than 75,000 chemicals (unique CAS-chemical name pairs) and more than 15,000 products (unique product names).

The CPDat data presented in the Dashboard is stored in a MySQL relational database maintained by the CPDat team. A copy of the MySQL database as of August 2017 is archived in FigShare (Data Citation 1). Users may install MySQL and download the data set (Data Citation 1) for manipulation and data extraction; however, users are encouraged to use the Dashboard as an access point, for ease of data manipulation and accessibility. Addition of new data, and updates to the underlying CPDat data set are ongoing, with data updates pushed to the public facing Dashboard periodically. Notification of updates will be posted on both the ‘News’ (https://comptox.epa.gov/dashboard/news_info) and ‘Downloads’ (https://comptox.epa.gov/dashboard/downloads) pages of the Dashboard, as appropriate. Details presented below represent data records archived in the MySQL database (Data Citation 1), and available on the Dashboard as of August 2017.

Within the MySQL CPDat database, two types of data are stored related to each data record: metadata, and record-specific data. Metadata refers to descriptions and information which relate to all, or to large subsets, of data records, as compared to record-specific data which is specific to a single data record (e.g., weight fraction of a chemical in a particular product). The mapping of the CASRN and/or chemical name provided by the data source to the DSSTox unique identifier underlies the presentation of data in the Dashboard itself. The ‘official’ CASRN, chemical name, and additional information about the chemical can be found on each chemical’s landing page within the Dashboard.

### Metadata

To ensure data provenance and tracking, metadata about each source the data records were obtained from has been linked to each data record. Metadata includes a brief description of the data source, the date the dataset was downloaded, and the web URL the data was downloaded from. Additionally, descriptions defining the product categorizations are reproduced in Table 2 (available online only). The specific metadata or definition associated with each data record is available in a pop-up window when hovering over or clicking on the data source name or the product categorization in associated tables in the Dashboard.

### Record-specific data

Record-specific data can include different pieces of information depending on the data source. All information provided by the original data source is included. A summary of the pieces of data included by data source is provided in Table 4. Record specific data available in the Dashboard may include:

#### Product name

Specific name of the product, as provided by the original data source (may or may not include brand name, or name of manufacturer).

#### Product use category

Product category assigned to the product, indicating the general category and product type assigned to each data record based on information provided in the original data source. Also see ‘Categorization type’ below.

#### Categorization type

Indicates the type of categorization assigned to the data record (CPCat Cassette or product category). Additional detail regarding CPCat Cassette categorizations can be found in Dionisio *et al.*^3^, detail regarding product categories can be found in Methods and Table 2 (available online only).

#### Minimum/Maximum weight fraction

For ‘MSDS’ data, the minimum and maximum weight fraction for each listed ingredient, as detailed on the associated MSDS. A minimum and maximum value is required because many MSDS report weight fraction as a range. Where an MSDS reported a single value, the minimum and maximum weight fraction is the same. For ‘Ingredient List’ data, the 5th and 95th percentiles of the predicted weight fraction (See Isaacs *et al.*^7^ for detail on how weight fractions were predicted based on ordered ingredient lists).

#### Data type

Form of product-specific data provided by original source (MSDS or Ingredient List).

#### Reported functional use

The functional use of the chemical in the product, as defined by the original data source.

#### Harmonized functional root

A simple abbreviation of a functional use allowing harmonization of different reporting names (e.g., ‘flavorant’ versus ‘flavouring’).

#### Harmonized functional use

The harmonized functional use, as determined by cluster analysis of the reported functions for each chemical. See ref. 5 for detailed methods.

#### Probability of harmonized functional use

Probability ranging from 0 to 1 that a chemical is likely to have one of the 41 harmonized functional uses with a valid QSUR model^6^.

#### Source

The original data source from which each specific data record was obtained.

The record-specific data available under each sub-tab of the ‘Exposure’ section on a chemical’s landing page in the Dashboard is summarized in Table 5.

## Technical Validation

The main quality objective for CPDat was ensuring that data included in the data set accurately reflected the data as provided in the raw data source. Efforts to curate the data set have not focused on checking for errors that may have been made by the original provider of the data (e.g., an error the manufacturer made in listing a weight fraction on a MSDS). Rather, quality assurance focused on ensuring that data records were transcribed accurately from the original data source and represented appropriately in the repository. A manual review of data (5% for Unilever USA MSDS, 1% for Proctor & Gamble MSDS) automatically extracted from PDF files was completed to ensure accuracy, with no errors identified. Note that for the Unilever USA MSDS data source, occasionally product names listed on the MSDS did not match the product name on the corresponding website link and in these cases, it was assumed that the MSDS contained the correct product name. In some cases, errors were identified in the process of migrating data from CSV files into the MySQL database (e.g., duplicated chemicals on a single product’s ingredient list). In these cases, custom Python scripts were written to check all data and correct for these errors.

The mappings of chemical records from CPDat to DSSTox unique chemical identifiers currently in use were obtained from a semi-automated mapping process. Within the semi-automated process, a baseline level of trust in the mapping must be met for the mapping to be maintained. Refinement of algorithms used in the semi-automated mapping process, and additional manual verification of mappings by a trained DSSTox curation team are always ongoing.

## Usage Notes

Though data included in CPDat can be used in many ways in future analyses, users should be aware of limitations of the data set, and appropriate usage of the data. Though a wide range of consumer products are included in CPDat, the products included in CPDat are necessarily limited to those for which data were publicly available. Thus, for example, personal care products are more heavily represented due to Food and Drug Administration reporting requirements for cosmetics. Additionally, each product category will contain multiple products, each with their own formulation. Though the range of different formulations within each product category allows the user to gain insight into similarities and differences (e.g., a particular chemical may be present in all formulations for a particular category), the formulations should not be assumed to be representative of all formulations, nor should they be assumed to accurately represent market share within a product category.

There are additional factors a user must consider, specifically when interpreting data obtained from MSDS. Formulations obtained from MSDS are associated with a date, representing the date the manufacturer issues the MSDS detailing composition of that particular formulation. Users should take care to note these associated dates and filter data as appropriate for their intended use since formulations of a product do change over time. Further, manufacturers are not required to report all ingredients in a product (only those which are potentially hazardous), although manufacturers may choose to report all ingredients. Quantitative composition information may also be withheld if the ingredient is considered a trade secret. Additionally, some chemicals and products are exempt from the hazard communication standards, e.g., solid articles or products covered under other legislation, such as foods, pharmaceuticals, and tobacco products^10^. Lastly, with the exception of a few data sources contained within CPCat, ingredients associated with product-specific records in CPDat are drawn from ingredient lists or MSDS, and by nature represent ‘intended’ ingredients in products. Therefore, CPDat does not currently capture the range of unknown contaminants which may be present in consumer products, for example, chemicals that may migrate from the plastic bottle of hand lotion into the hand lotion itself.

CPDat includes predictions of functional use based on machine-learning classification models developed from chemical structures^6^. As with any QSAR method, these predictions are by their nature limited by the depth and breadth of the chemical space of the training set on which the classification models rely. Any use of these predictions should consider these limitations in evaluating their fitness-for-purpose. The training set used to develop the models is composed of the reported function data (also included in CPDat); the modeling methodology and model performance has been previously discussed in detail^6^.

## Additional information

**How to cite this article**: Dionisio, K. L. *et al*. The Chemical and Products Database, a resource for exposure-relevant data on chemicals in consumer products. *Sci. Data* 5:180125 doi: 10.1038/sdata.2018.125 (2018).

**Publisher’s note**: Springer Nature remains neutral with regard to jurisdictional claims in published maps and institutional affiliations.

## Supplementary Material



## Figures and Tables

**Table 1:** Additional data sources included in CPDat (beyond the CPCPdb, CPCat, and FUse data sets).

Data source	Description	URL	Download date	Data included	Collected files	CPDat product records^a^
Unilever	Manufacturer including multiple leading brands of personal care and household products	https://pioti.unilever.com/PIOTI/EN/p2.asp	7/17/2015	Product specific, weight fraction-ordered ingredient lists and functional use disclosures	948	846
Unilever MSDS USA	Manufacturer including multiple leading brands of personal care and household products	http://www.unilevermsdsusa.com/	10/8/2015	Quantitative product composition	577	282
Proctor & Gamble	Manufacturer including multiple leading brands of personal care and household products	http://www.pgproductsafety.com/productsafety/sds/SDS_2015/	9/19/2015	Quantitative product composition	2403	1799
Proctor & Gamble Product Safety	Manufacturer including multiple leading brands of personal care and household products	http://www.pg.com/productsafety/search_results.php?searchtext=*&submit=Search&submit=Search	7/15/2015	Product-specific ingredient and functional use disclosures	250	226
Drugstore.com	Online retail site for personal care and household products. Website no longer functional.	http://www.drugstore.com/	9/1/2015	Product-specific ingredient lists	4635	4635
Church & Dwight	Manufacturer including multiple leading brands of personal care and household products	http://www.churchdwight.com/brands-and-products/msds-ingredient-search.aspx	7/16/2015	Product-specific ingredient and functional use disclosures	35	34
Palmolive	Single brand website for liquid dish soaps	http://www.palmolive.com/ingredients#soft-touch-aloe	7/17/2015	Product-specific ingredient and functional use disclosures	16	16

^a^Database record count varies from collected files due to removal of products with no listed ingredients or no manufacturer-provided CASRN, duplicated products, and multi-component products.

**Table 2:** Product categories.

Unique ID	General Category	Product Type	Refined Product Type	Description
P.AC.010.029	arts and crafts	arts and crafts adhesive	spray	spray adhesives for primarily craft purposes, including spray mounts and stencil placement sprays
P.AC.010.099	arts and crafts	arts and crafts adhesive	NA	glue sticks, glitter glues, fabric glues, craft glue, and other adhesives used for primarily craft purposes
P.AC.020.099	arts and crafts	arts and crafts cleaner	NA	solvent-based products for cleaning paint, adhesives, etc. from hands or surfaces
P.AC.030.029	arts and crafts	arts and crafts finish	spray	spray shellacs or polyurethane coatings for primarily craft purposes
P.AC.030.099	arts and crafts	arts and crafts finish	NA	shellacs or polyurethane coatings for primarily craft purposes
P.AC.040.099	arts and crafts	arts and crafts paint	NA	paints and colorants for primarily craft purposes (including acrylic and enamel paints)
P.AC.050.029	arts and crafts	body paint	spray	body paints, markers, glitters, play cosmetics, and halloween cosmetics (spray or aerosol formulation specified)
P.AC.050.099	arts and crafts	body paint	NA	body paints, markers, glitters, play cosmetics, and halloween cosmetics
P.AC.060.099	arts and crafts	bubble solution	NA	liquid bubble solutions, including solutions for bubble machines
P.AC.070.099	arts and crafts	craft kit	NA	multi-component crafting kits where individual products are not designated
P.AC.080.099	arts and crafts	crayons	NA	wax crayons for coloring or illustration
P.AC.090.099	arts and crafts	dye	NA	products for dying fabrics
P.AC.100.029	arts and crafts	fabric paints and sealers	spray	paints or sealers for treating fabrics (spray or aerosol formulation specified)
P.AC.100.099	arts and crafts	fabric paints and sealers	NA	paints or sealers for treating fabrics
P.AC.110.099	arts and crafts	finger paint	NA	non-edible finger paints
P.AC.120.029	arts and crafts	flocking	spray	flocking and artificial snow (spray or aerosol formulation specified)
P.AC.130.099	arts and crafts	fogger	NA	liquid solutions for fogger machines
P.AC.140.099	arts and crafts	glaze	NA	liquid roducts for glazing craft pottery
P.AC.150.099	arts and crafts	gun cleaner	NA	liquid solutions for cleaning interior or exterior of firearms
P.AC.160.099	arts and crafts	pens and markers	NA	writing utensils containing liquid or gel ink
P.AC.170.099	arts and crafts	play dough	NA	children's play modeling clays
P.AP.010.099	auto products	antifreeze	NA	antifreeze and/or coolant solutions for motorized vehicles
P.AP.020.029	auto products	auto air freshener	spray	air fresheners for car interiors (spray or aerosol formulation specified)
P.AP.020.099	auto products	auto air freshener	NA	air fresheners for car interiors, including products for vents
P.AP.030.029	auto products	auto fluids and additives	spray	power steering fluids, transmission fluids, brake fluids, fuel injector cleaners, gas treatments, or leak stoppers (spray or aerosol formulation specified)
P.AP.030.099	auto products	auto fluids and additives	NA	power steering fluids, transmission fluids, brake fluids, fuel injector cleaners, gas treatments, or leak stoppers
P.AP.040.029	auto products	auto lubricant	spray	engine lubricants and belt dressings, not including motor oils (spray or aerosol formulation specified)
P.AP.040.099	auto products	auto lubricant	NA	engine lubricants and belt dressings, not including motor oils
P.AP.050.029	auto products	auto paint	spray	paints and primers for auto body or engine (spray or aerosol formulation specified)
P.AP.050.099	auto products	auto paint	NA	paints and primers for auto body or engine
P.AP.060.099	auto products	auto refrigerant	NA	refrigerants and freon products for auto applications
P.AP.070.099	auto products	boat cleaner	NA	cleaners, washes, and polishes for exterior marine applications
P.AP.080.099	auto products	boat engine fluids	NA	engine fluids for marine applications
P.AP.090.099	auto products	body cleaner	NA	cleaners, shampoos, and washes for auto body exterior (not including wax products)
P.AP.100.029	auto products	body repair	spray	products for repairing auto body exteriors, including bondo-type products and scratch fillers (spray or aerosol formulation specified)
P.AP.100.099	auto products	body repair	NA	products for repairing auto body exteriors, including bondo-type products and scratch fillers
P.AP.110.029	auto products	body wax	spray	auto body waxes and coatings, including combo wash/wax products (spray or aerosol formulation specified)
P.AP.110.099	auto products	body wax	NA	auto body waxes and coatings, including combo wash/wax products
P.AP.120.029	auto products	degreaser	spray	auto, engine and brake degreasers (spray or aerosol formulation specified)
P.AP.120.099	auto products	degreaser	NA	auto, engine and brake degreasers
P.AP.130.029	auto products	detailing	spray	products for cleaning, polishing, or protecting car interior surfaces, apholstery, leather, carpeting, tires, or rims (spray or aerosol formulation specified
P.AP.130.099	auto products	detailing	NA	products for cleaning, polishing, or protecting car interior surfaces, apholstery, leather, carpeting, tires, or rims
P.AP.140.099	auto products	motor oil	NA	petroleum-based or synthetic engine lubricants
P.AP.150.029	auto products	windows/windshield	spray	products for washing or protecting windshields or windows (spray or aerosol formulation specified)
P.AP.150.099	auto products	windows/windshield	NA	products for washing or protecting windshields or windows
P.HM.010.029	home maintenance	adhesive remover	spray	solvent products for removing adhesives from surfaces (spray or aerosol formulation specified)
P.HM.010.099	home maintenance	adhesive remover	NA	solvent products for removing adhesives from surfaces
P.HM.020.099	home maintenance	caulk/sealant	NA	caulks and sealers for household use, including silicone products
P.HM.030.099	home maintenance	concrete	NA	products for patching or cleaning concrete surfaces
P.HM.040.029	home maintenance	corrosion protection	spray	products for protecting metal surfaces from corrosion or removing corrosion (spray or aerosol formulation specified)
P.HM.040.099	home maintenance	corrosion protection	NA	products for protecting metal surfaces from corrosion or removing corrosion
P.HM.050.099	home maintenance	degreaser	NA	products for degreasing indoor surfaces
P.HM.060.029	home maintenance	finish	spray	products for permanently coating and protecting wood surfaces, including polyurethane products (spray or aerosol formulation specified)
P.HM.060.099	home maintenance	finish	NA	products for permanently coating and protecting wood surfaces, including polyurethane products
P.HM.070.099	home maintenance	grout sealer	NA	products for coating and protecting tile or grout
P.HM.080.099	home maintenance	lock deicer	NA	products for deicing car or residential locks
P.HM.090.029	home maintenance	lubricant	spray	household maintenance lubricants (spray or aerosol formulation specified)
P.HM.090.099	home maintenance	lubricant	NA	household maintenance lubricants
P.HM.100.099	home maintenance	mortar or grout	NA	products for attaching and grouting interior tiles
P.HM.110.029	home maintenance	multipurpose adhesive	spray	general purpose repair adhesives including all purpose glues, super glue, and epoxies; not including wood glues (spray or aerosol formulation specified)
P.HM.110.099	home maintenance	multipurpose adhesive	NA	general purpose repair adhesives including all purpose glues, super glue, and epoxies; not including wood glues
P.HM.120.005	home maintenance	paint	exterior	home improvement paints characterized as exterior use
P.HM.120.009	home maintenance	paint	interior	home improvement paints characterized as interior use
P.HM.120.029	home maintenance	paint	spray	home improvement paints not characterized as to interior or exterior use (spray or aerosol formulation specified)
P.HM.120.099	home maintenance	paint	NA	home improvement paints not characterized as to interior or exterior use
P.HM.130.029	home maintenance	paint cleaner	spray	paint removing products for clean-up (spray or aerosol formulation specified)
P.HM.130.099	home maintenance	paint cleaner	NA	paint removing products for clean-up
P.HM.140.099	home maintenance	paint texture	NA	textured paints including ceiling texture
P.HM.150.099	home maintenance	paint thinner	NA	paint or lacquer thinners
P.HM.160.099	home maintenance	patch and repair	NA	drywall, ceiling, and joint compounds, spackles, or fillers
P.HM.170.099	home maintenance	plumbing	NA	miscellaneous plumbing repair products (putties, tapes, cements)
P.HM.180.005	home maintenance	primer	exterior	home improvement primers characterized as exterior use
P.HM.180.009	home maintenance	primer	interior	home improvement primers characterized as interior use
P.HM.180.029	home maintenance	primer	spray	home improvement primers not characterized as to interior or exterior use (spray or aerosol formulation specified)
P.HM.180.099	home maintenance	primer	NA	home improvement primers not characterized as to interior or exterior use
P.HM.190.099	home maintenance	putty or filler	NA	putty-type crack, hole, and wood fillers
P.HM.200.099	home maintenance	refrigerant	NA	air conditioner or other refrigerants
P.HM.210.099	home maintenance	roof	NA	products for roof maintenance or repair, including cleaners, sealers, or coatings
P.HM.220.099	home maintenance	septic system	NA	septic system treatment products
P.HM.230.099	home maintenance	spray foam	NA	spray insulation and weatherstripping products
P.HM.240.005	home maintenance	stain	exterior	wood stains characterized as exterior use, including deck stains
P.HM.240.099	home maintenance	stain	NA	wood stains not characterized as interior or exterior use
P.HM.250.029	home maintenance	stripper	spray	paint and finish strippers (spray or aerosol formulation specified)
P.HM.250.099	home maintenance	stripper	NA	paint and finish strippers
P.HM.260.005	home maintenance	surface sealer	exterior	products for coating and protecting household surfaces characterized as exterior use
P.HM.260.029	home maintenance	surface sealer	spray	products for coating and protecting household surfaces not characterized as interior or exterior use (spray or aerosol formulation specified)
P.HM.260.099	home maintenance	surface sealer	NA	products for coating and protecting household surfaces not characterized as interior or exterior use
P.HM.270.099	home maintenance	welding	NA	miscellaneous welding products including gases, fluxes, and adhesives
P.HM.280.099	home maintenance	wood adhesive	NA	adhesives specifically characterized as wood glues
P.HO.010.099	home office	printer ink	NA	ink for inkjet printers
P.HO.020.099	home office	printer toner	NA	laser printer toners
P.HO.030.099	home office	white out	NA	correction fluids for ink or type
P.IH.010.007	inside the home	air freshener	gel	home air fresheners (gel formulation specified)
P.IH.010.013	inside the home	air freshener	liquid	home air fresheners (liquid formulation specified)
P.IH.010.016	inside the home	air freshener	oil or diffuse	home air fresheners (oil formulations or diffusers)
P.IH.010.029	inside the home	air freshener	spray	home air fresheners (spray or aerosol formulation specified)
P.IH.010.099	inside the home	air freshener	NA	home air fresheners
P.IH.020.099	inside the home	automatic dishwashing additive	NA	rinse aids, spot removers, and dishwasher cleaners
P.IH.030.007	inside the home	automatic dishwashing detergent	gel	detergents for automatic dishwashers (gel formulation specified)
P.IH.030.023	inside the home	automatic dishwashing detergent	powder	detergents for automatic dishwashers (powder formulation specified)
P.IH.030.099	inside the home	automatic dishwashing detergent	NA	detergents for automatic dishwashers
P.IH.040.029	inside the home	bathroom cleaner	spray	bathtub, tile, and toilet surface cleaners (spray or aerosol formulation specified)
P.IH.040.099	inside the home	bathroom cleaner	NA	bathtub, tile, and toilet surface cleaners
P.IH.050.099	inside the home	bleach	NA	bleaches (including color-safe bleaches)
P.IH.060.099	inside the home	candles	NA	candles
P.IH.070.029	inside the home	carpet cleaner	spray	carpet cleaning products and machine solutions (spray or aerosol formulation specified)
P.IH.070.099	inside the home	carpet cleaner	NA	carpet cleaning products and machine solutions
P.IH.080.099	inside the home	carpet deodorizer	NA	carpet deodorizers
P.IH.090.099	inside the home	dish soap	NA	hand dish washing liquids and detergents
P.IH.100.029	inside the home	disinfectant	spray	disinfecting liquids (spray or aerosol formulation specified)
P.IH.100.099	inside the home	disinfectant	NA	disinfecting liquids (including wipes)
P.IH.110.099	inside the home	drain	NA	drain openers or digesters
P.IH.120.099	inside the home	dry cleaner	NA	dry cleaning fluids or kits for home use
P.IH.130.099	inside the home	dryer sheets	NA	dryer fabric softener sheets
P.IH.140.029	inside the home	electronics cleaner	spray	miscellaneous products for cleaning home electronic equipment (including surface cleaners or cleaners for interior parts) (spray or aerosol formulation spec
P.IH.140.099	inside the home	electronics cleaner	NA	miscellaneous products for cleaning home electronic equipment (including surface cleaners or cleaners for interior parts)
P.IH.150.099	inside the home	fabric deodorizer	NA	spray fabric deodorizers
P.IH.160.099	inside the home	fabric protectant	NA	fabric protectants and wrinkle releasers
P.IH.170.099	inside the home	fabric softener	NA	fabric softeners (excluding dryer sheets)
P.IH.180.099	inside the home	fireplace	NA	miscellaneous fireplace and firepit products
P.IH.190.099	inside the home	floor cleaner	NA	hard floor cleaners (including premoistened wipes and pads)
P.IH.200.099	inside the home	floor polish	NA	hard floor shining and waxing products
P.IH.210.029	inside the home	glass cleaner	spray	glass and window cleaners (spray or aerosol formulation specified)
P.IH.210.099	inside the home	glass cleaner	NA	glass and window cleaners
P.IH.220.099	inside the home	hand cleaner	NA	products marketed as hand cleaners (solvent-based)
P.IH.230.029	inside the home	heavy duty cleaner	spray	heavy duty or concentrated multipurpose cleaners (spray or aerosol formulation specified)
P.IH.230.099	inside the home	heavy duty cleaner	NA	heavy duty or concentrated multipurpose cleaners
P.IH.240.029	inside the home	houseplant care	spray	houseplant and cut flower foods, fertilizers, or pesticides (spray or aerosol formulation specified)
P.IH.240.099	inside the home	houseplant care	NA	houseplant and cut flower foods, fertilizers, or pesticides
P.IH.250.099	inside the home	lamp oil/lighter fluid	NA	lighters, lighter fluids, and other indoor fuels
P.IH.260.013	inside the home	laundry detergent	liquid	laundry detergents and soaps (liquid formulation specified)
P.IH.260.023	inside the home	laundry detergent	powder	laundry detergents and soaps (powder formulation specified)
P.IH.260.099	inside the home	laundry detergent	NA	laundry detergents and soaps
P.IH.270.099	inside the home	laundry fragrance	NA	scent products to be added to wash
P.IH.280.029	inside the home	laundry stain remover	spray	stain removers or laundry pre-treatment products (spray or aerosol formulation specified)
P.IH.280.099	inside the home	laundry stain remover	NA	stain removers or laundry pre-treatment products
P.IH.290.029	inside the home	laundry starch	spray	fabric starches (spray or aerosol formulation specified)
P.IH.290.099	inside the home	laundry starch	NA	fabric starches
P.IH.300.099	inside the home	lime remover	NA	products for removing lime or scale from surfaces
P.IH.310.099	inside the home	metal polish	NA	cleaning products for metal surfaces
P.IH.320.029	inside the home	oven cleaner	spray	oven and grill cleaners (spray or aerosol formulation specified)
P.IH.320.099	inside the home	oven cleaner	NA	oven and grill cleaners
P.IH.330.099	inside the home	shoe polish or protectant	NA	shoe polishes, cleaners, and protectants
P.IH.340.029	inside the home	surface cleaner	spray	hard surface and kitchen surface cleaners (spray or aerosol formulation specified)
P.IH.340.099	inside the home	surface cleaner	NA	hard surface and kitchen surface cleaners
P.IH.350.099	inside the home	upholstery cleaner	NA	upholstery fabric cleaners
P.IH.360.029	inside the home	wood polish	spray	wood or furniture dusting or polishing products (spray or aerosol formulation specified)
P.IH.360.099	inside the home	wood polish	NA	wood or furniture dusting or polishing products
P.LY.010.005	landscape/yard	cleaner	exterior	exterior surface (e.g. deck, house, driveway) cleaners
P.LY.020.099	landscape/yard	garden care	other	miscellaneous garden care products not otherwise characterized (e.g. rooting powders)
P.LY.030.099	landscape/yard	garden fertilizer	NA	fertilizers for vegetable or flower gardens, including in combination with pest/weed controllers
P.LY.040.099	landscape/yard	grill/camping fuel	NA	grill, lantern and camping stove fuels, including briquets
P.LY.050.029	landscape/yard	herbicide	spray	herbicides, weed killers, and brush killers (spray or aerosol formulation specified)
P.LY.050.099	landscape/yard	herbicide	NA	herbicides, weed killers, and brush killers
P.LY.060.099	landscape/yard	lawn fertilizer	NA	fertilizers for lawns, including in combination with pest/weed controllers
P.LY.070.099	landscape/yard	lawnmower fluids	NA	oils and other fluids for lawnmowers or other small yard care equipment
P.LY.080.099	landscape/yard	mulch	NA	mulches, including those with weed controllers
P.LY.090.002	landscape/yard	pool chemicals	algaecide	algaecidal products for pools, hot tubs, and spas
P.LY.090.003	landscape/yard	pool chemicals	chlorinating	chlorinating products for pools, hot tubs, and spas
P.LY.090.022	landscape/yard	pool chemicals	ph control	ph control products for pools, hot tubs, and spas
P.LY.090.025	landscape/yard	pool chemicals	shock	shock products for pools, hot tubs, and spas
P.LY.090.099	landscape/yard	pool chemicals	NA	products not otherwise characterized for pools, hot tubs, and spas
P.LY.100.099	landscape/yard	potting soil	NA	potting soil and vermiculite
P.LY.110.099	landscape/yard	surface deicer	NA	products for deicing surfaces
P.LY.120.099	landscape/yard	trees	NA	miscellaneous tree care products including sealers
P.PC.010.029	personal care	acne spot treatment	spray	creams and wipes for spot-treating acne (spray or aerosol formulation specified
P.PC.010.099	personal care	acne spot treatment	NA	creams and wipes for spot-treating acne
P.PC.020.099	personal care	aftershave	NA	lotions, balms, liquids, and gels for post-shave applications (usually fragranced)
P.PC.030.099	personal care	baby lotion	NA	baby cream or lotion (excluding diaper creams)
P.PC.040.099	personal care	baby oil	NA	skin oils specifically marketed for babies
P.PC.050.099	personal care	baby powder	NA	powders specifically marketed for babies
P.PC.060.099	personal care	baby shampoo	NA	shampoos specifically marketed for babies
P.PC.070.099	personal care	baby wash	NA	body washes and cleaners specifically marketed for babies
P.PC.080.099	personal care	baby wipes	NA	diaper and other baby wipes
P.PC.090.099	personal care	bar soap	NA	bar and other solid soaps
P.PC.100.099	personal care	bath oil	NA	bath oils or oil-filled beads
P.PC.110.099	personal care	bath paints/crayons	NA	bath paints or bath crayons
P.PC.120.099	personal care	bath salts	NA	bath salts, soaks, and fizzes
P.PC.130.099	personal care	bite relief	NA	soothing treatments for insect bites
P.PC.140.099	personal care	blush/bronzer	NA	cheek blushes, bronzers, and rouges
P.PC.150.099	personal care	body adhesive	NA	NA
P.PC.160.099	personal care	body care set	NA	multicomponent body care or bath set for which individual products are not designated
P.PC.170.029	personal care	body oil	spray	body oils (not including baby or bath oils, spray or aerosol formulation specified)
P.PC.170.099	personal care	body oil	NA	body oils (not including baby or bath oils)
P.PC.180.099	personal care	body powder	NA	talcum and dusting powders for the body
P.PC.190.099	personal care	body scrub	NA	body cleaners containing abrasives or exfoliants
P.PC.200.099	personal care	body wash	NA	body cleaners, washes, shower gels
P.PC.210.099	personal care	body wipes	NA	body wipes and towelettes (excluding diaper or baby wipes)
P.PC.220.099	personal care	bubble bath	NA	bubble baths
P.PC.230.099	personal care	clipper lubricant/cleaner	NA	cleaning and lubricating products for hair clippers
P.PC.240.029	personal care	contact care	spray	contact lense cleaners and solutions (spray or aerosol formulation specified)
P.PC.240.099	personal care	contact care	NA	contact lense cleaners and solutions
P.PC.250.099	personal care	cosmetic tool cleaner	NA	NA
P.PC.260.099	personal care	denture adhesive	NA	denture adhesives
P.PC.270.099	personal care	denture cleaner	NA	denture cleansers
P.PC.280.007	personal care	deodorant	gel	deodorants and antiperspirants (gel formulation specified)
P.PC.280.028	personal care	deodorant	solid	deodorants and antiperspirants (solid formulation specified)
P.PC.280.029	personal care	deodorant	spray	deodorants and antiperspirants (spray or aerosol formulation specified)
P.PC.280.099	personal care	deodorant	NA	deodorants and antiperspirants not otherwise characterized
P.PC.290.029	personal care	depilatory	spray	chemical products for removal of body or facial hair (spray or aerosol formulation specified)
P.PC.290.099	personal care	depilatory	NA	chemical products for removal of body or facial hair
P.PC.300.099	personal care	diaper cream	NA	diaper creams and ointments
P.PC.310.029	personal care	dry shampoo	spray	dry shampoos for removing hair oils (spray or aerosol formulation specified)
P.PC.310.099	personal care	dry shampoo	NA	dry shampoos for removing hair oils
P.PC.320.099	personal care	ethnic hair care	NA	products for ethnic hair care, including wrap set lotions and pressing lotions (excluding relaxers)
P.PC.330.099	personal care	eye cream	NA	creams and moisturizers for specific treatment of eye area
P.PC.340.099	personal care	eye drops	NA	products for lubricating eyes or treating redness
P.PC.350.029	personal care	eye lid spray	spray	NA
P.PC.360.013	personal care	eye liner	liquid	eye liners or brow coloring products (liquid or gel formulation specified)
P.PC.360.099	personal care	eye liner	NA	eye liners or brow coloring products
P.PC.370.099	personal care	eye makeup	other	miscellaneous cosmetic eye products including lash adhesives and tints
P.PC.380.099	personal care	eye products	other	NA
P.PC.390.099	personal care	eye shadow	NA	products for coloring eye lids
P.PC.400.099	personal care	face cleansing wipes	NA	textile wipes or pads treated with cleansing solution
P.PC.410.029	personal care	face cream/moisturizer	spray	moisturizers, lotions, and creams for primarily treating the face (excluding eye-specific products) (spray or aerosol formulation specified)
P.PC.410.099	personal care	face cream/moisturizer	NA	moisturizers, lotions, and creams for primarily treating the face (excluding eye-specific products)
P.PC.420.099	personal care	face mask	NA	leave-on masks or peels for treatment of the face
P.PC.430.099	personal care	face powder	NA	pressed or loose powders for face
P.PC.440.001	personal care	face scrub	acne	facial cleansing products containing exfoliating particles, for acne treatment
P.PC.440.099	personal care	face scrub	NA	facial cleansing products containing exfoliating particles (excluding products for acne)
P.PC.450.001	personal care	face wash	acne	facial cleansing products (excluding scrubs), for acne treatment
P.PC.450.029	personal care	face wash	spray	facial cleansing products (excluding scrubs) (spray or aerosol formulation specified)
P.PC.450.099	personal care	face wash	NA	facial cleansing products (excluding scrubs and products for acne)
P.PC.460.029	personal care	foot care	spray	miscellaneous products for application to feet, including scrubs, lotions, and deodorants (spray or aerosol formulation specified)
P.PC.460.099	personal care	foot care	NA	miscellaneous products for application to feet, including scrubs, lotions, and deodorants
P.PC.470.099	personal care	foundation/concealer	NA	liquid or cream foundation make-ups and concealers
P.PC.480.029	personal care	fragrance	spray	fragrances, colognes, and perfumes (spray or aerosol formulation specified)
P.PC.480.099	personal care	fragrance	NA	fragrances, colognes, and perfumes
P.PC.490.099	personal care	glitter	NA	powder products containing reflective particles for face or body
P.PC.500.099	personal care	hair bleach	NA	products for lightening or removing color from hair on the head
P.PC.510.019	personal care	hair color	permanent	hair colors and dyes characterized as permanent
P.PC.510.024	personal care	hair color	professional	hair colors and dyes characterized as for professional use
P.PC.510.030	personal care	hair color	temporary	hair colors and dyes characterized as temporary
P.PC.510.031	personal care	hair color	temporary|spra	hair colors and dyes characterized as temporary (spray or aerosol formulation specified)
P.PC.510.099	personal care	hair color	NA	hair coloring products not otherwise categorized
P.PC.520.099	personal care	hair color activator	NA	chemical activators for hair coloring products
P.PC.530.099	personal care	hair color developer	NA	chemical developers for hair coloring products
P.PC.540.099	personal care	hair color toner	NA	chemical toners for hair coloring products
P.PC.550.011	personal care	hair conditioner	leave-in	leave-in everyday hair conditioners and detanglers
P.PC.550.012	personal care	hair conditioner	leave-in|spray	leave-in everyday hair conditioners (spray or aerosol formulation specified)
P.PC.550.029	personal care	hair conditioner	spray	rinse-out everyday hair conditioners (excluding combo shampoo/conditioner products) (spray or aerosol formulation specified)
P.PC.550.099	personal care	hair conditioner	NA	rinse-out everyday hair conditioners (excluding combo shampoo/conditioner products)
P.PC.560.024	personal care	hair conditioning treatment	professional	hair conditioning and moisturizing treatments for occasional use characterized as for professional use
P.PC.560.029	personal care	hair conditioning treatment	spray	hair conditioning and moisturizing treatments for occasional use (spray or aerosol formulation specified)
P.PC.560.099	personal care	hair conditioning treatment	NA	hair conditioning and moisturizing treatments for occasional use
P.PC.570.099	personal care	hair relaxer	NA	chemical hair relaxers
P.PC.580.099	personal care	hair spray	NA	spray fixatives for hair
P.PC.590.007	personal care	hair styling	gel	hair styling products for hold, shine, or texture (gel formulation indicated)
P.PC.590.008	personal care	hair styling	gel|spray	hair styling products for hold, shine, or texture (gel and spray formulation indicated)
P.PC.590.014	personal care	hair styling	mousse	foaming hair styling products
P.PC.590.015	personal care	hair styling	mousse|spray	NA
P.PC.590.023	personal care	hair styling	powder	NA
P.PC.590.029	personal care	hair styling	spray	hair styling products for hold, shine, or texture (spray or aerosol formulation specified)
P.PC.590.099	personal care	hair styling	NA	hair styling products for hold, shine, or texture
P.PC.600.029	personal care	hand sanitizer	spray	antibacterial products for hands (spray or aerosol formulation specified)
P.PC.600.099	personal care	hand sanitizer	NA	antibacterial products for hands
P.PC.610.099	personal care	hand soap	NA	liquid hand soaps
P.PC.620.099	personal care	hand wipes	NA	wipes and solution primarily for hands
P.PC.630.007	personal care	hand/body lotion	gel	lotions and creams primarily for hands and body (gel formulations indicated)
P.PC.630.008	personal care	hand/body lotion	gel|spray	lotions and creams primarily for hands and body (gel and spray or aerosol formulation specified)
P.PC.630.029	personal care	hand/body lotion	spray	lotions and creams primarily for hands and body (spray or aerosol formulation specified)
P.PC.630.099	personal care	hand/body lotion	NA	lotions and creams primarily for hands and body
P.PC.640.099	personal care	lice shampoo	NA	shampoos for the treatment of head lice
P.PC.650.099	personal care	liniment	NA	liniments and ointments for treatment of muscle or joint pain
P.PC.660.099	personal care	lip balm	NA	lip products primarily for protection
P.PC.670.099	personal care	lip color	NA	colored lip products, excluding glosses
P.PC.680.099	personal care	lip gloss	NA	glossy lip products
P.PC.690.099	personal care	lip liner	NA	pencils for lining lips
P.PC.700.099	personal care	makeup primer	NA	products for priming face for make-up
P.PC.710.099	personal care	makeup remover	NA	products for removing face make-ups
P.PC.720.099	personal care	makeup set	NA	multicomponent make-up set set for which individual products are not designated
P.PC.730.099	personal care	mascara	NA	eyelash mascaras
P.PC.740.029	personal care	mouthwash	spray	antiseptic and dental mouthwashes and rinses (spray or aerosol formulation specified)
P.PC.740.099	personal care	mouthwash	NA	antiseptic and dental mouthwashes and rinses
P.PC.750.099	personal care	nail adhesive	NA	adhesives for reparing fingernails or attaching artificial nails
P.PC.760.099	personal care	nail polish	NA	clear or colored nail enamels, polishes, basecoats, topcoats, and other acrylic coatings
P.PC.770.099	personal care	nail polish remover	NA	products for removing nail polish or artificial nails
P.PC.780.018	personal care	nail products	other|spray	miscellaneous products for nail or cuticle treatment not otherwise characterized (spray or aerosol formulation specified)
P.PC.780.099	personal care	nail products	other	miscellaneous products for nail or cuticle treatment not otherwise characterized
P.PC.790.029	personal care	scalp treatment	spray	products for treating the scalp, including products dandruff or hair loss (spray or aerosol formulation specified)
P.PC.790.099	personal care	scalp treatment	NA	products for treating the scalp, including products dandruff or hair loss
P.PC.800.029	personal care	self-tanner	spray	chemical products for tanning, staining, or coloring the skin (spray or aerosol formulation specified)
P.PC.800.099	personal care	self-tanner	NA	chemical products for tanning, staining, or coloring the skin
P.PC.810.099	personal care	sexual wellness	NA	sexual wellness products, including personal lubricants
P.PC.820.004	personal care	shampoo	dandruff	shampoos, including dual shampoo/conditioner products for treatment of dandruff
P.PC.820.099	personal care	shampoo	NA	shampoos, including dual shampoo/conditioner products
P.PC.830.007	personal care	shaving cream	gel	shaving creams, foams, balms and soaps (gel formulation specified)
P.PC.830.099	personal care	shaving cream	NA	shaving creams, foams, balms and soaps
P.PC.840.029	personal care	sunscreen	spray	suncreens and blocks (spray or aerosol formulations specified
P.PC.840.099	personal care	sunscreen	NA	suncreens and blocks
P.PC.840.100	personal care	sunscreen	children	suncreens and blocks marketed to children or babies
P.PC.840.101	personal care	sunscreen	children|spray	suncreens and blocks marketed to children or babies (spray or aerosol formulations specified
P.PC.850.099	personal care	teeth whitener	NA	teeth whitening products (excluding toothpastes)
P.PC.860.099	personal care	toner	NA	face and skin toners and astringents
P.PC.870.007	personal care	toothpaste	gel	toothpastes and dentrifices (gel formulation specified)
P.PC.870.099	personal care	toothpaste	NA	toothpastes and dentrifices
P.PC.880.099	personal care	waxing	NA	wax products for removing hair from face or body
P.PE.010.029	pesticides	animal repellent	spray	chemical animal repellents (spray or aerosol formulation specified)
P.PE.010.099	pesticides	animal repellent	NA	chemical animal repellents
P.PE.020.029	pesticides	fungicide	spray	fungicides for garden or home use (spray or aerosol formulation specified)
P.PE.020.099	pesticides	fungicide	NA	fungicides for garden or home use
P.PE.030.005	pesticides	insect repellent	exterior	miscellaneous products for repelling insects, exterior use indicated
P.PE.030.006	pesticides	insect repellent	exterior|spray	miscellaneous products for repelling insects, exterior use indicated (spray or aerosol formulation specified)
P.PE.030.026	pesticides	insect repellent	skin	products for repelling insects from skin
P.PE.030.027	pesticides	insect repellent	skin|spray	products for repelling insects from skin (spray or aerosol formulation specified)
P.PE.030.029	pesticides	insect repellent	spray	miscellaneous products for repelling insects (excluding products to be applied to skin) (spray or aerosol formulation specified)
P.PE.030.099	pesticides	insect repellent	NA	miscellaneous products for repelling insects (excluding products to be applied to skin)
P.PE.040.005	pesticides	insecticide	exterior	insecticides, exterior use indicated - including lawn and gardens
P.PE.040.006	pesticides	insecticide	exterior|spray	insecticides, exterior use indicated (spray or aerosol formulation specified)
P.PE.040.009	pesticides	insecticide	interior	insecticides, interior use indicated
P.PE.040.010	pesticides	insecticide	interior|spray	insecticides, interior use indicated (spray or aerosol formulation specified)
P.PE.040.029	pesticides	insecticide	spray	insecticides not otherwise characterized (spray or aerosol formulation specified)
P.PE.040.099	pesticides	insecticide	NA	insecticides not otherwise characterized
P.PE.050.099	pesticides	rodenticide	NA	rodenticides for interior or exterior use
P.PT.010.099	pet care	aquarium	NA	miscellaneous aquarium treatment products
P.PT.020.099	pet care	cat litter	NA	cat litters
P.PT.030.029	pet care	other pet treatments	spray	miscellaneous pet treatments (excluding pesticides) (spray or aerosol formulation specified)
P.PT.030.099	pet care	other pet treatments	NA	miscellaneous pet treatments (excluding pesticides)
P.PT.040.020	pet care	pesticide	pet	pesticides for application to pets
P.PT.040.021	pet care	pesticide	pet|spray	pesticides for application to pets (spray or aerosol formulation specified)
P.PT.050.099	pet care	pet shampoo	NA	pet shampoos (inclduing those contain pesticides)
P.PT.060.029	pet care	pet stain cleaner	spray	carpet and upholstery cleaners for pet stains (spray or aerosol formulation specified)
P.PT.060.099	pet care	pet stain cleaner	NA	carpet and upholstery cleaners for pet stains

**Table 3:** Performance of QSUR Models for Function Used to Develop Predictions in CPDat.

Harmonized Function	Model Misclassification Error	Y-randomization Error (Error for a model with randomized descriptors - will be higher than misclassification error for a valid model)	5-fold Cross-Validation Error	Balanced Accuracy	Valid Model?	Descriptor Set Providing Best Performance
additive	0.046754331	0.200849509	0.04847192	0.917611326	1	structural and physicochemical descriptors
adhesion_promoter	0.023168441	0.195721144	0.023314903	0.940745732	1	structural and physicochemical descriptors
antimicrobial	0.084324776	0.138674598	0.111417376	0.832231369	1	structural and physicochemical descriptors
antioxidant	0.125652265	0.185656439	0.141470757	0.830642433	1	structural and physicochemical descriptors
antistatic_agent	0.0845335	0.195827593	0.087158136	0.957460184	1	structural and physicochemical descriptors
buffer	0.100605302	0.199574202	0.10281037	0.877372942	1	structural and physicochemical descriptors
catalyst	0.076393237	0.178893759	0.09295391	0.844362042	1	structural and physicochemical descriptors
chelator	0.060799173	0.251262487	0.059657578	0.927744079	1	structural descriptors
colorant	0.065957003	0.154297641	0.093573354	0.86152553	1	structural and physicochemical descriptors
crosslinker	0.065748278	0.181292006	0.076394618	0.854983694	1	structural and physicochemical descriptors
emollient	0.067835525	0.184151534	0.074956938	0.877650181	1	structural and physicochemical descriptors
emulsifier	0.085994573	0.186927573	0.089767299	0.956903766	1	structural and physicochemical descriptors
emulsion_stabilizer	0.128315536	0.247180503	0.123594786	0.887990287	1	structural descriptors
film_forming_agent	0.102066374	0.188975162	0.11561325	0.850119119	1	structural and physicochemical descriptors
flame_retardant	0.049467752	0.189415571	0.057835208	0.911195989	1	structural and physicochemical descriptors
flavorant	0.201515673	0.296789528	0.225449232	0.802825413	1	structural descriptors
foam_boosting_agent	0.026868756	0.234981054	0.029516291	0.961519184	1	structural descriptors
foamer	0.039958663	0.238646228	0.05404652	0.888814809	1	structural descriptors
fragrance	0.104362346	0.091312878	0.180354306	0.833370385	0	structural and physicochemical descriptors
hair_conditioner	0.108745565	0.185840117	0.115556867	0.8723559	1	structural and physicochemical descriptors
hair_dye	0.083706511	0.296481226	0.086465375	0.913851078	1	structural descriptors
heat_stabilizer	0.095595909	0.196121895	0.103383064	0.819908898	1	structural and physicochemical descriptors
liquid_system_additive	0.213744402	0.242810885	0.212867404	0.665048239	0	structural descriptors
lubricating_agent	0.094557354	0.234307613	0.097420918	0.852726018	1	structural descriptors
masking_agent	0.265931795	0.290768171	0.268045014	0.706807656	0	structural descriptors
monomer	0.030056356	0.187562096	0.033261089	0.955749957	1	structural and physicochemical descriptors
organic_pigment	0.042197726	0.290506373	0.045864148	0.95336717	1	structural descriptors
oxidizer	0.104030313	0.237537031	0.11108987	0.906210448	1	structural descriptors
perfumer	0.238154874	0.181221039	0.248188991	0.778019277	0	structural and physicochemical descriptors
photoinitiator	0.059421288	0.23329659	0.064523303	0.845120898	1	structural descriptors
plasticizer	0.181364106	0.250235963	0.189032958	0.821963434	0	structural descriptors
preservative	0.150490503	0.195537466	0.156851135	0.762548364	1	structural and physicochemical descriptors
reducer	0.075767063	0.195896473	0.080884551	0.899384817	1	structural and physicochemical descriptors
rheology_modifier	0.05781674	0.233414736	0.054891898	0.740139099	0	structural and physicochemical descriptors
rubber_additive	0.038408543	0.24304857	0.042802803	0.830676329	1	structural descriptors
skin_conditioner	0.116259654	0.177163431	0.128201895	0.816574896	1	structural and physicochemical descriptors
skin_protectant	0.088290545	0.198933417	0.097833786	0.787421384	1	structural and physicochemical descriptors
soluble_dye	0.057354461	0.244688254	0.059253575	0.970623812	1	structural descriptors
solvent	0.154873722	0.19057608	0.168262119	0.794776513	0	structural and physicochemical descriptors
surfactant	0.061991234	0.175971613	0.069677574	0.932168925	1	structural and physicochemical descriptors
ubiquitous	0.24922494	0.295277299	0.268400274	0.698813401	0	structural descriptors
UV_absorber	0.106441612	0.282466414	0.11411815	0.852169052	1	structural descriptors
vinyl	0.015654352	0.196731371	0.016493576	0.99214166	1	structural and physicochemical descriptors
viscosity_controlling_agent	0.196827385	0.20435191	0.195650275	0.680335711	0	structural and physicochemical descriptors
wetting_agent	0.02359628	0.249638305	0.024620005	0.946486222	1	structural descriptors
whitener	0.026696521	0.231906648	0.026814662	0.941171857	1	structural descriptors
For details see Phillips *et al*^6^.						

**Table 4:** Data records included in CPDat as of August 8, 2017.

	Product use categorization		Record-specific data		
Source	CPCat cass.	Prod. Cat.	Weight fraction	Ingredient list	Functional use
CPCat	X				
CPCPdb		X	X		
FUse	NA	NA			X
Unilever		X		X	X
Unilever MSDS USA		X	X		
Proctor & Gamble		X	X		
Proctor & Gamble Product Safety		X		X	X
Drugstore.com		X		X	
Church & Dwight		X		X	X
Palmolive		X		X	X

**Table 5:** Record specific data fields available under each sub-tab of the ‘Exposure’ section of the Dashboard.

Product & Use Category sub-tab	Chemical Weight Fraction sub-tab	Chemical Functional Use sub-tab
Product or Use categorization	Product name	Harmonized Functional Use
Categorization type	Product category	Reported Functional Use
Number of unique products	Minimum weight fraction	Probability of Associated Functional Use
	Maximum weight fraction	
	Data type	
	Source	
